# Dyspepsia and Indigestion

**Published:** 1885-05

**Authors:** 


### Dyspepsia and Indigestion.

Dr. Austin Flint is arousing the prophets of common sense to the management of the stomach, and the endeavor to overcome its difficulties. He is beginning to recognize that there is a physiological fallacy in the idea that what is called weak digestion, or inability to take certain articles of food, is a malady which the sufferer ought either accept as a dispensation of Providence, or to meet by uncompromising surrender. This is how Dr. Austin Flint puts the matter. "The mind plays an important part in the etiology of the affection. The old method of treatment was to strictly regulate the diet, drink little or no fluids, and always leave the table hungry. Such treatment is entirely wrong; dyspepsia may be developed by the attempt to regulate the diet by rules intended to prevent the affection. I always ask a patient, "do you regulate your diet?" and he always answers in the affirmative. I have never known a dyspeptic to get well who attempted to regulate his diet. Regulate by the appetite, by the palate, and by common sense. A patient may ask, am I not to be guided by personal experience, and avoid such articles as I have found to disagree with me? I answer that personal experience is often deceptive. An article that would disagree to-day would agree to-morrow. Do not adopt the rules of eating only twice or thrice a day; be governed by the appetite. Those articles are most digestible which are most acceptible to the palate. Do not leave the table hungry; take animal and vegetable products and drink according to the want of instinct. The diet which in healthy subjects is conducive to health is the best diet for dyspeptics. It is a fallacy to suppose that in dyspepsia the stomach needs a long rest. Patients need not be afraid to rely on their digestive powers. Perfect cures have been obtained by following the instincts of nature. Dyspepsia is more common among the educated classes because they try to regulate their diet on scientific principles."

There is much sound sense in this view; it is not new, but only the old-fashioned, sensible, manly one revived. Physicians, some at least who have not posed as "starving doctors," or as anti-alcohol and anti-tobacco practitioners, have been striving to stem the course of fanaticism in science, but vainly. Perhaps it was not worth while to struggle and toil against the stream. Those who have laid aside silently on the bank, waiting until the tide turned, have had a less troubled time of it; and now since it has turned, or is rapidly turning, doubtless more from natural causes than from efforts made by opponents of the "fad" of fashionable physicians. It will be amusing to see how the advanced fanatics change their policy. Already we hear it admitted that "port is a good sustaining wine." Before long it may be perceived that, since the days when good sound wine was recognized as a useful as well as permissible aid to digestion, and in itself a serviceable nutrient, the type of many maladies has been altered, and not for the better; while bad nervous affections and weaknesses, neuralgia, throat maladies, and a host of distressing and debilitating disorders have been notably on the increase; while as regards the *bete noire* of the anti-constitutionalist—another development of the fad in physic—the gout, a suppressed or undeveloped form of the disorder, has replaced the genuine old-fashioned enemy, against which the weapons of art were infinitely more serviceable than they are wont to prove against the bronchitic troubles, the liver, kidney, heart, brain and nerve disturbances which have sprung into prominence as the type of the manifestation of the gout has been reduced—or shall we say depressed—by the withdrawal of stimulants. Dr. Austin Flint's remarks appear opportunely during the delivery and publication of Dr. Lauder Brunton's instructive lectures before the Medical Society of London.—*British Medical Journal.*

---

Dr. Dureau de Villeneuve, in a note presented by M. Marey to the Academie des Sciences of Paris, recommends that distilled water should be generally used for drinking purposes. He argues that pure water is a desideratum, especially during cholera epidemics. Distilled water fulfills this condition, and could be easily obtained by utilizing the condensers of the different steam engines kept at works. Dr. Villeneuve suggests that water thus distilled should be distributed among the inhabitants of the different districts near the seat of distillation.

